# Endoscopic Versus Open Resection for Small Gastric Gastrointestinal Stromal Tumors: Safety and Outcomes

**DOI:** 10.1097/MD.0000000000000376

**Published:** 2015-01-09

**Authors:** Chaoyong Shen, Haining Chen, Yuan Yin, Jiaju Chen, Luyin Han, Bo Zhang, Zhixin Chen, Jiaping Chen

**Affiliations:** From the Department of Gastrointestinal Surgery (CS, HC, YY, JC, BZ, ZC, JC); and Intensive Care Unit (LH), West China Hospital, Sichuan University, Chengdu, Sichuan, China.

## Abstract

Endoscopic resection has been performed to treat small gastric neoplasms. However, this technique for small gastric gastrointestinal stromal tumors (GISTs) remains controversial. This study aims to compare the safety and surgical outcomes of endoscopic versus open resection of small gastric GISTs.

The medical records of 54 consecutive gastric GISTs patients with tumor size of ≤2 cm, who were surgically treated with endoscopic resection (endoscopic group) or open surgery (laparotomy group) in a single institution from March 2010 to June 2014, were retrospectively analyzed. The clinical and tumor characteristics, surgical safety, and tumor-related outcomes were evaluated.

Of 54 patients, 32 and 22 patients underwent endoscopic resection and laparotomy, respectively. Patients who underwent endoscopic resection yielded a significantly shorter hospital stay compared with patients who underwent laparotomy (*P* < 0.001). Compared with patients in the endoscopic group, patients in the laparotomy group had more intraoperative blood loss (*P* < 0.001), had longer nasogastric tube retention (*P* < 0.001), and required longer operative time (*P* < 0.001). More laparotomy patients required postoperative analgesic drugs than those in the endoscopic group (n = 9 vs 4; *P* = 0.016). Gastric perforation occurred in 1 case during operation in the endoscopic group. Patients who underwent these 2 procedures did not differ with respect to tumor size (*P* = 0.168), perioperative transfusion (*P* = 1.000), reoperation (*P* = 1.000), early satiety (*P* = 0.560), and postoperative bleeding (*P* = 1.000). With a median follow-up time of 34.5 months, 1 high-risk patient in each group experienced tumor recurrence/metastasis postoperatively.

The endoscopic procedure allows safe resection with good surgical outcomes for small gastric GISTs compared with laparotomy. Moreover, larger randomized controlled trials are warranted to confirm endoscopic application for small gastric GISTs.

## INTRODUCTION

Gastrointestinal stromal tumors (GISTs), which originate from the interstitial cells of Cajal or its precursor, may be asymptomatic and nonmalignant when diagnosed but have a potential for malignant transformation.^1,2^ Currently, the therapeutic guideline of small GISTs as identified by endoscopic ultrasonography (EUS) remains uncertain. Some small masses gradually grow, may have symptoms, and finally undergo malignant transformation.^3,4^ Thus, surgical treatment becomes necessary. Macroscopically and histologically negative surgical margins and avoidance of tumor rupture are the principles of surgical treatment.^5^ However, previous reports have shown that recurrence of GISTs was primarily dependent on tumor biology rather than microscopic margins.^6^ Thus, various types of surgical procedures, such as traditional open surgery, laparoscopic and endoscopic resection, have been performed for GISTs.

To date, several publications have reported endoscopic resection techniques, including endoscopic submucosal dissection (ESD),^7,8^ submucosal tunneling endoscopic resection,^9^ and ligation-assisted endoscopic enucleation.^10,11^ Findings from recent reports have demonstrated that in patients with GISTs, even in tumors with a maximum size of up to 5 cm, endoscopic resection is safe and feasible.^12^ Theoretically, endoscopic resection is simple and feasible for some tumors, but the risk of early tumor recurrence caused by incomplete excision has become a major concern for surgeons. By contrast, open surgery, which can remove the entire tumor with a histologically negative margin, is widely recognized as the most effective procedure to treat malignant tumors from both technical and oncologic points of view. However, to the best of our knowledge, although previous case series have reported endoscopic resection for GISTs, the safety and surgical outcomes between endoscopic and open resection of GISTs have not yet been reported. We, therefore, set out to assess the outcomes of endoscopic resection in comparison to the traditional abdominal surgical approach for the treatment of small gastric GISTs.

## MATERIALS AND METHODS

### Patient Selection

Patients with gastric stromal tumors who were not randomly treated with endoscopic resection or laparotomy in a single medical institution (West China Hospital, Sichuan University, Sichuan, China) between March 2010 and June 2014 were reviewed. Operation consents were obtained from each patient in this cohort. However, the institutional review board and committee of the West China Hospital of Sichuan University deemed that the approval of the committee was not required for the retrospective analysis of clinical data. The following patients were included in the study: patients with tumor diameter not larger than 2 cm based on preoperative EUS and/or abdominal computerized tomography (CT) examination; patients who had not taken aspirin, warfarin, or other nonsteroidal anti-inflammatory drug for at least 1 week before the endoscopic resection; patients who had normal complete blood count, prothrombin time, and thrombin time; patients with no other malignant tumors; and patients who were pathologically diagnosed as having GISTs preoperatively or postoperatively. Patients who refused surgical intervention were excluded. The surgical approaches were decided according to the tumor growth pattern, EUS findings, or patients’ preference.

### Surgical Procedures and Management

**FIGURE 1:**
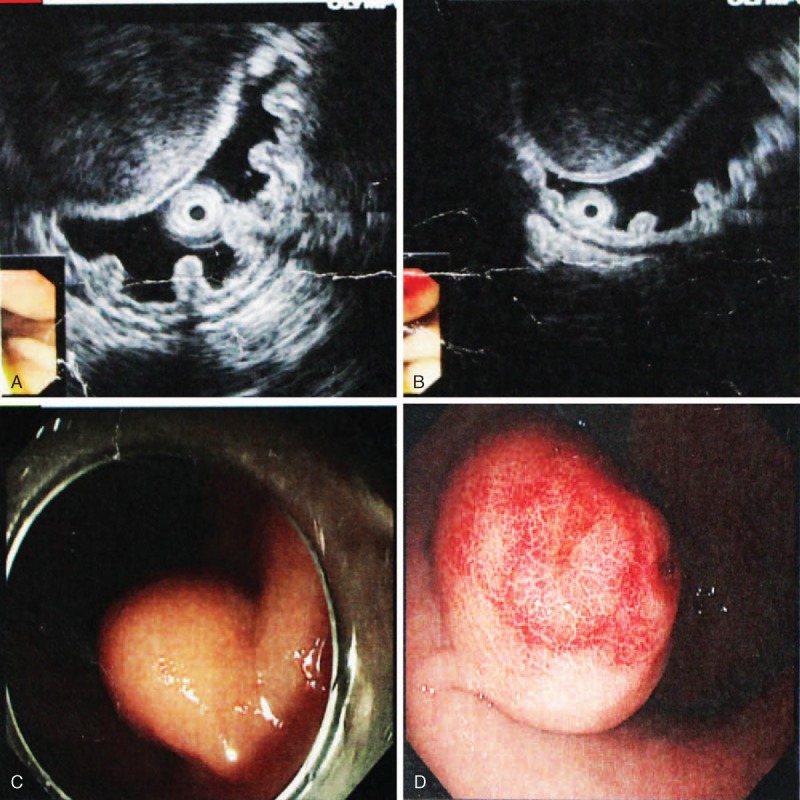
(A and B) Images of lesions located in the muscularis propria of the middle part of the stomach with a fairly clear boundary, as confirmed by EUS. (C and D) Endoscopic view of tumors located in the gastric body and tumors growing with an intragastric-type pattern. EUS = endoscopic ultrasonography.

**FIGURE 2:**
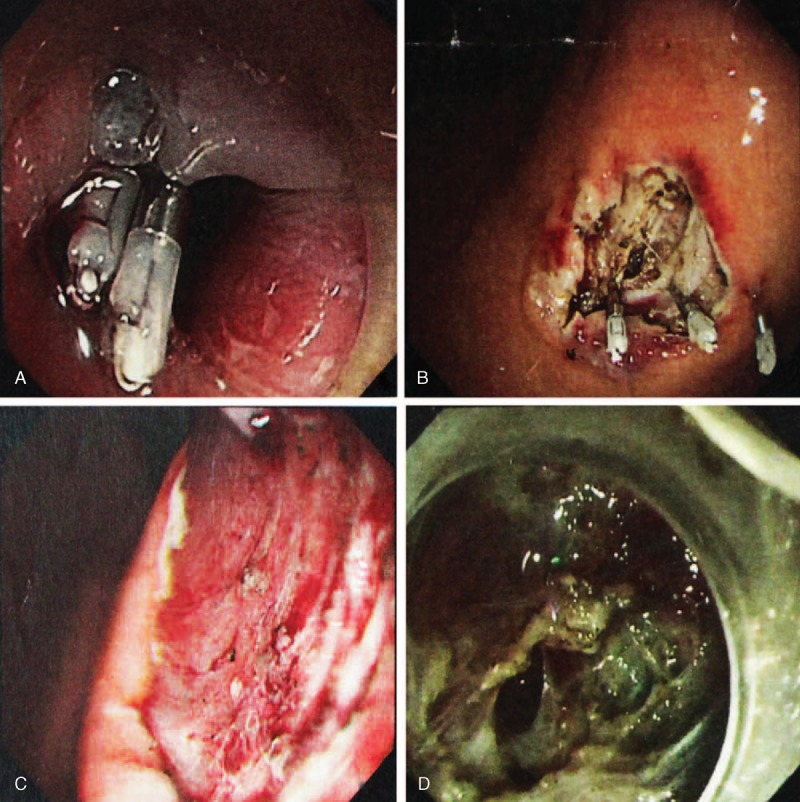
(A and B) Images of wound surfaces were closed with titanium clips after excision of lesion. (C) Endoscopic view of wound surface tumor; no tumor tissue was left under the endoscopic inspection. (D) Gastric perforation occurred during the operation because of obvious adhesion to the serosal layer of the stomach.

All patients received general anesthesia. The surgical procedures included ESD (endoscopic group) and open resection (laparotomy group). Endoscopic resection was mainly chosen for patients with tumors originating from the muscularis propria or tumors with intragastric type (Figure 1A–D) and clear boundaries to adjacent tissues and organs. All endoscopic resections were performed by skilled endoscopic specialists and surgeons. For patients in the endoscopic group, EUS was again performed to determine the tumor size, layer of origin, and tumor growth pattern before the procedure. A hook knife, insulated-tip knife, needle knife, grasping forceps, and titanium clips were used to dissect the tumor. First, we submucosally injected methylene blue-stained saline, including epinephrine (1:100 000), to lift the lesion off the muscle layer. A hook knife or insulated-tip knife was used to cut the mucosal surface and dissect the tumor. Electrocautery snare and norepinephrine were used in some cases to reduce bleeding. The wound was closed with titanium clips, and medical adhesive was used for some patients to immobilize clips and healing wounds (Figure 2A and B). Nasogastric tube was routinely retained after the procedure. Endoscopic complete resection of tumors is regarded as the absence of residual tumor tissue macroscopically on endoscopy (Figure 2C) and microscopically. All patients received proton-pump inhibitors, and endoscopic examinations were performed postoperatively. For patients in the laparotomy group, the procedure started with a traditional midline upper abdominal incision. Frozen slices of the incisal margin and tumor were routinely collected during surgery. The operation was regarded as completed once the surgical margins were confirmed to be negative. Patients with diabetes were insulinized preoperatively and medications were adjusted to rational control hypertension. A soft diet was allowed for patients with ESD who were free from perforation or endoscopic bleeding, and food was allowed for laparotomy patients after removal of gastrointestinal decompression.

### Data Collection and Postoperative Follow-Up

The parameters measured that were retrospectively reviewed from their medical records included demographic and clinicopathological characteristics, surgical data (procedures, intraoperative findings, operative times, and complications), morbidity, mortality, and duration of hospital stay. Follow-up was regularly conducted through office visit, telephone, or outpatient clinic visit from July 2014 to August 2014. During the follow-up, data collection included surgical complications, postoperative adjuvant therapy with imatinib mesylate (Glivec/Gleevec; Novartis Pharma AG, Basel, Switzerland), tumor recurrence/metastasis, and death. EUS and abdominal CT scanning were used every 6 to 12 months postoperatively.

### Statistical Analysis

Median values were used to describe continuous data. Quantitative results were expressed as mean ± standard deviation. Categorical data from different groups were compared using the χ^2^ test or Fisher exact test. Wilcoxon test was used to test ranked data. Differences between the 2 groups were compared using an independent sample *t* test. All data analyses were performed using SPSS version 17.0 (SPSS, Chicago, IL) statistical software package for Microsoft Windows. *P* values of <0.05 were considered to be statistically significant.

## RESULTS

### Demographic Data and Clinical Characteristics

Table 1 summarizes the clinical and demographic features between 2 surgical approaches. All 54 patients with small gastric GISTs underwent endoscopic resection or surgical resection through the abdominal approach, and 15 patients were diagnosed with GIST preoperatively. The endoscopic group (n = 32) was composed of 15 males (46.9%) and 17 females (53.1%) and had a median age of 61 years (range, 37–81 years). Accordingly, the laparotomy group was composed of 11 males (50.0%) and 11 females (50.0%), with a median age of 55 years (range, 37–73 years). The most common clinical symptom was abdominal pain or discomfort, followed by alimentary tract bleeding. Patients in the endoscopic group had a significantly shorter hospital stay than the patients in the laparotomy group (5.34 ± 2.34 vs 13.68 ± 3.52 days; *P* < 0.001). The gender, age, and history of diabetes or hypertension did not differ between either of the groups.

**TABLE 1:**
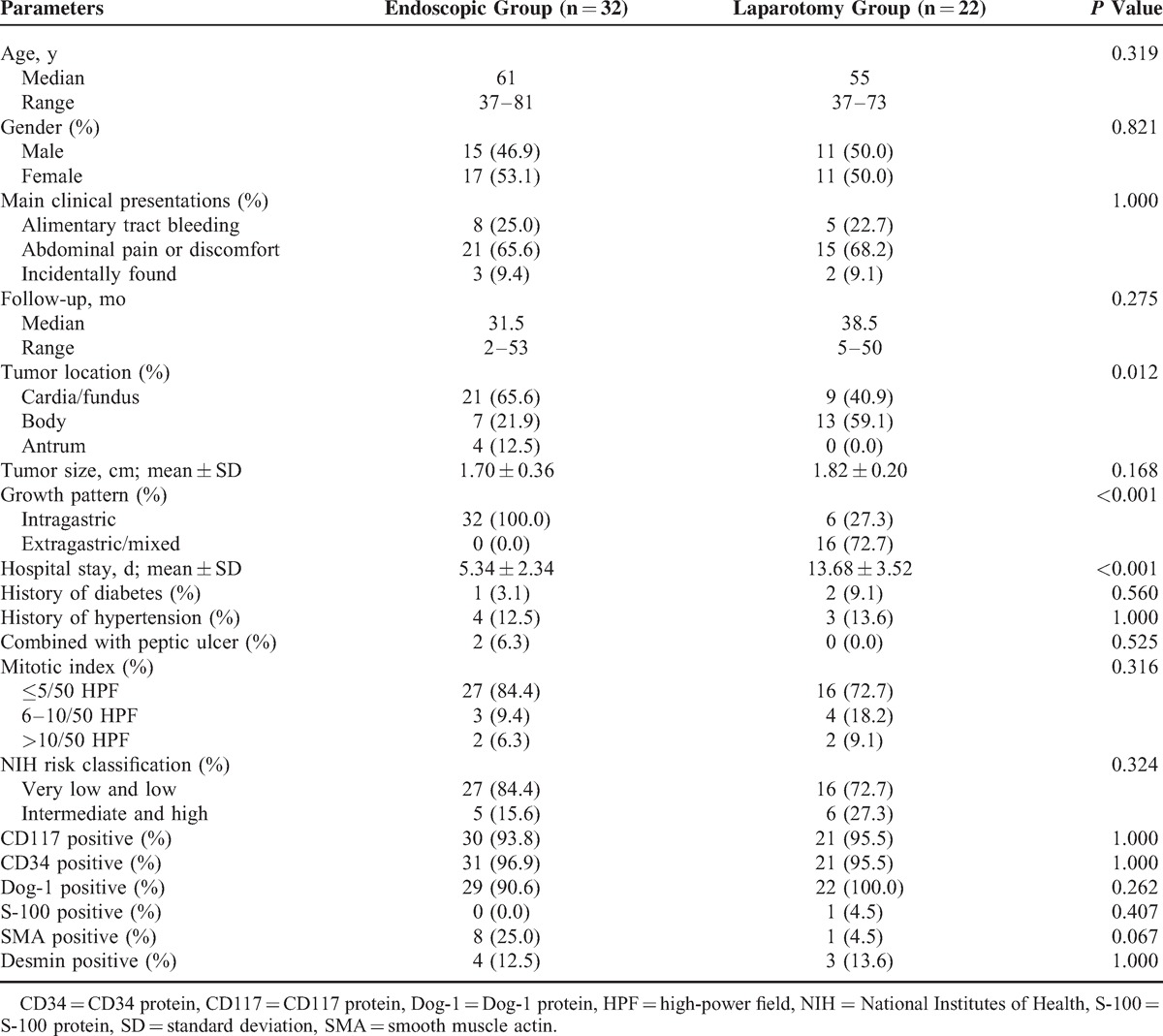
Clinicopathologic and Demographic Features for Gastric Gastrointestinal Stromal Tumors Between 2 Surgical Approaches

### Tumor Characteristics

A total of 30 masses were located in the cardia or fundus, 20 in the body, and 4 in the antrum. The endoscopic group had a higher proportion of tumors located in the cardia/fundus compared with that of the laparotomy group (*P* = 0.012). A significant difference was observed in the growth patterns between the 2 groups (*P* < 0.001). According to the modified National Institutes of Health (NIH) risk categories,^13^ in the endoscopic group, 9 tumors were of very low risk, 18 were of low risk, 3 were of intermediate risk, and 2 were of high risk. In the laparotomy group, 3 tumors were of very low risk, 13 were of low risk, 4 were of intermediate risk, and 2 were of high risk. A total of 5 patients with intermediate/high risk were treated with imatinib mesylate (400 mg/d). No significant differences were found between the endoscopic and the laparotomy group with respect to mitotic count (*P* = 0.316), NIH risk classification (*P* = 0.324), tumor size (*P* = 0.168), and immunohistochemical characteristics (*P* > 0.05).

### Surgical Information

None of the patients underwent total or subtotal gastrectomy. Open surgery with local resection was performed in all 22 cases, whereas 32 patients underwent ESD. Compared with patients in the endoscopic group, patients had more intraoperative blood loss (7.38 ± 4.25 vs 24.09 ± 6.38 mL; *P* < 0.001), had longer nasogastric tube retention times (2.16 ± 0.92 vs 4.32 ± 1.76; *P* < 0.001), and required longer operative time (31.34 ± 10.24 vs 56.36 ± 11.97 min; *P* < 0.001) in the laparotomy group. The extragastric or mixed lesions were more likely to be treated with laparotomy (*P* < 0.001). Only 1 patient in the endoscopic group underwent perioperative transfusion because of massive hemorrhage of gastrointestinal tract preoperatively, as shown in Table 2.

**TABLE 2:**
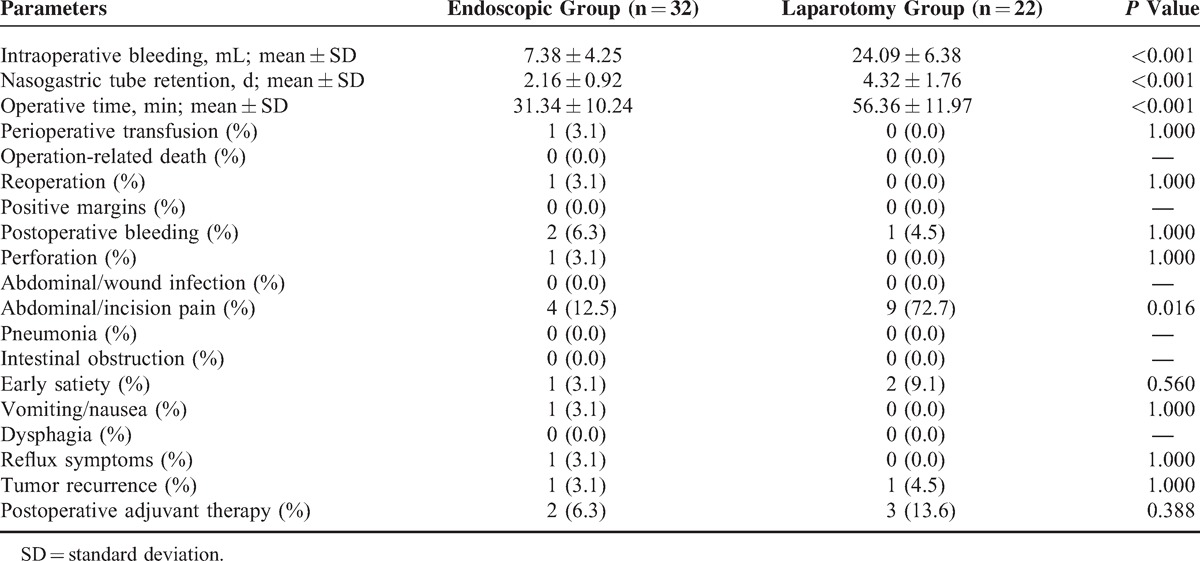
Operative Information and Postoperative Complications Between the Endoscopic and the Laparotomy Groups

### Postoperative Complications

No significant differences were found between the 2 groups with respect to reoperation (*P* = 1.000), early satiety (*P* = 0.560), and postoperative bleeding (*P* = 1.000). No surgery-related deaths occurred in either group during the perioperative period. However, 1 episode of gastric perforation occurred during operation in the endoscopic group, and laparoscopic repair was conducted (Figure 2D). This patient had no evidence of tumor recurrence or distant metastasis with a 13-month follow-up postoperatively. More patients required postoperative analgesic drugs in the laparotomy group than those in the endoscopic group because of abdominal or incisional pain (n = 9 vs 4; *P* = 0.016). In the endoscopic group, 1 patient had vomiting/nausea, and 1 patient had gastroesophageal reflux symptoms. Of these patients, only 1 required a proton-pump inhibitor.

### Outcomes

Follow-up was achieved in all 54 patients, with a median follow-up of 34.5 months (31.5 months in the endoscopic group; 38.5 months in the laparotomy group). One patient at high risk in each group experienced recurrence or liver metastasis at 23 and 31 months postoperatively. One patient with endoscopic resection died 18 months after hospital discharge because of chronic respiratory failure. No patient died because of progression of the GISTs.

## DISCUSSIONS

In this study, we focused on the safety and outcomes between ESD and open resection for small gastric GISTs (tumor size ≤2 cm). This study is important because we reviewed our experiences and assessed the safety and effectiveness of endoscopic resection for small gastric GIST cases. We demonstrate that patients who underwent ESD had less intraoperative blood loss, less nasogastric tube retention time, less operative time, and fewer patients who required postoperative analgesic drugs compared with open surgery, although it cannot totally avoid perforation during the procedure. Moreover, no obvious postoperative tumor recurrence or metastasis occurred in the ESD group. In summary, our data suggest that ESD allows safe resection with good surgical outcomes for small GISTs.

GISTs are the most common mesenchymal tumors of the gastrointestinal tract with an estimated incidence of 10 to 20 per million, and more than half of GISTs are located in the stomach.^3,14^ All GISTs have malignant potential; however, this potential may vary depending on tumor size and mitotic activity.^15,16^ The true malignant potential cannot be accurately evaluated by noninvasive auxiliary examination. Recurrence or metastasis may be observed for tumors with low mitotic count or small-sized tumors. Therefore, all GISTs, including small ones, are recommended for surgical resection once they are diagnosed.^17^ Small GISTs are usually asymptomatic or only combined with slight abdominal discomfort and can be found incidentally by endoscopy or abdominal CT scanning.^18^ A preoperative pathological biopsy diagnosis for GISTs is sometimes needed. However, biopsy procedures are difficult for small tumors, especially those only several millimeters in size.^19^

Traditionally, complete resection, avoidance of tumor rupture, and achievement of R0 resection should be obeyed during operation.^16^ Currently, with the advances in endoscopic devices and techniques, minimally invasive surgical resection including endoscopic, laparoscopic, and combined techniques has been performed widely in the treatment of gastric GISTs.^12,20^ Compared with open surgery, laparoscopic resection for gastric GISTs has been proven to be feasible and safe, whereas its application to small tumors is limited, especially in tumors <10 mm.^21,22^ Endoscopic resection has more advantages for elderly patients or patients with small-sized tumors; however, it remains controversial because of major complications, such as significant bleeding and perforation. Furthermore, the inherent risk of tumor spillage and positive microscopic margins is a huge concern.^23^ As such, long-term or even lifelong follow-up is needed if the tumor was removed by using a minimally invasive procedure. By contrast, open surgery can remove the entire lesion as well as a part of the normal tissue, which can provide a firmly negative surgical margin. However, an increasing number of studies have recently demonstrated that endoscopic resection can be safely performed with acceptable postoperative complications in selected cases.^10,12,24^ Given that many unanswered questions and equivocal evidence remain concerning the role of endoscopic resection for small gastric GISTs, we conducted this study to assess the feasibility of the endoscopic procedure.

Our series of patients who underwent endoscopic or laparotomy resection of gastric GISTs did not differ with respect to age, gender, tumor size, mitotic index, and clinicopathologic characteristics. However, patients with endoscopic resection had a higher proportion of tumors located in the cardia/fundus. As previously reported, endoscopic resection is less invasive than laparoscopic or open surgical interventions. However, the quick recovery, short postoperative hospitalization, and reduced amount of intraoperative bleeding for endoscopic procedure were noted.^11,12^ The procedure time, nasogastric tube retention time, and intraoperative bleeding were significantly lower for endoscopic resection than in the laparoscopic intervention in this cohort. Generally, perforation cannot be avoided during the resection, even if performed by an experienced specialist,^8,12,25,26^ especially for GISTs tightly adherent to the muscularis propria or tumors with extragastric growth. In this series, 1 patient who underwent endoscopy experienced a perforation; however, the patient did not suffer from severe peritoneal cavity inflammation. Finally, gastric perforation was effectively repaired by laparoscopic procedure. Bleeding is also a common complication after surgery; nevertheless, severe postoperative bleeding sometimes needs reoperation. Our data showed that bleeding occurred in 2 patients in the endoscopic group and in 1 patient in the laparotomy group; however, all these patients were relieved after conservative medical therapy. Small GISTs normally do not recur after surgical removal of the tumors.^9,10,24^ The follow-up results from the 2 groups were comparable. With a median follow-up of 34.5 in this cohort, 2 patients with high risk in each group were found to have tumor recurrence or liver metastasis based on EUS examination or abdominal CT scans. No patients died because of GISTs according to our observations, although 1 patient in the endoscopic group died of respiratory failure. Of note, patients in the laparotomy group suffered from more pain compared with patients in the endoscopic resection group. Open surgery needs longer operation time and undergoes more invasive procedure compared with other procedures.

Perforation and bleeding may occur after endoscopic resection; thus, several measures were applied for wound closure of GISTs. Hemoclips, which were developed by Japanese scholars, have been used to manage various types of perforations and fistulas^11,27^ and can also effectively prevent bleeding. Tissue adhesives were frequently used during endoscopic resection, including fibrin sealant, cyanoacrylates, and thrombin.^28,29^ Cyanoacrylates have primarily been used in the treatment of bleeding from gastric varices with excellent results, and fibrin sealant is usually utilized for the closure of gastrointestinal fistula. Within the limitations of an observational and limited number of patients, our study has shown that endoscopic resection of gastric GISTs should be limited in selected cases. Long-term follow-up should be performed to assess the safety and effectiveness of endoscopic resection of GISTs. Therefore, our findings should be verified by multicenter and randomized controlled trials in the future.

In conclusion, few studies have compared the endoscopic and open approaches for small GISTs. Endoscopic procedure allows for safe resection with good surgical outcomes for small GISTs, although it cannot totally avoid perforations and bleeding during the procedure.
